# Engineering nanoparticles to overcome barriers to immunotherapy

**DOI:** 10.1002/btm2.10005

**Published:** 2016-06-20

**Authors:** Randall Toy, Krishnendu Roy

**Affiliations:** ^1^ Wallace H. Coulter Dept. of Biomedical Engineering Georgia Institute of Technology, and Emory University Atlanta GA 30332

**Keywords:** cancer immunotherapy, drug delivery, intracellular delivery, targeted nanoparticles, tissue permeation, vaccines

## Abstract

Advances in immunotherapy have led to the development of a variety of promising therapeutics, including small molecules, proteins and peptides, monoclonal antibodies, and cellular therapies. Despite this wealth of new therapeutics, the efficacy of immunotherapy has been limited by challenges in targeted delivery and controlled release, that is, spatial and temporal control on delivery. Particulate carriers, especially nanoparticles have been widely studied in drug delivery and vaccine research and are being increasingly investigated as vehicles to deliver immunotherapies. Nanoparticle‐mediated drug delivery could provide several benefits, including control of biodistribution and transport kinetics, the potential for site‐specific targeting, immunogenicity, tracking capability using medical imaging, and multitherapeutic loading. There are also a unique set of challenges, which include nonspecific uptake by phagocytic cells, off‐target biodistribution, permeation through tissue (transport limitation), nonspecific immune‐activation, and poor control over intracellular localization. This review highlights the importance of understanding the relationship between a nanoparticle's size, shape, charge, ligand density and elasticity to its vascular transport, biodistribution, cellular internalization, and immunogenicity. For the design of an effective immunotherapy, we highlight the importance of selecting a nanoparticle's physical characteristics (e.g., size, shape, elasticity) and its surface functionalization (e.g., chemical or polymer modifications, targeting or tissue‐penetrating peptides) with consideration of its reactivity to the targeted microenvironment (e.g., targeted cell types, use of stimuli‐sensitive biomaterials, immunogenicity). Applications of this rational nanoparticle design process in vaccine development and cancer immunotherapy are discussed.

## 1 Introduction

Immunotherapy is a burgeoning field that holds promise for making an impact in the treatment of incurable disorders, for example, cancer, HIV, emerging infectious diseases, inflammatory diseases, and autoimmune disorders. A wide range of therapeutic modalities have been developed to regulate immunity, which include vaccines (e.g., melanoma gp‐100), recombinant cytokines (e.g., GM‐CSF, IL‐7, IL‐12), monoclonal antibodies (e.g., anti‐CTLA4, anti‐PD1), autologous T‐cells, and small molecules designed for specific intracellular targets (e.g., IDO1 inhibitors, COX2 inhibitors, Toll‐like receptor [TLR] agonists).^1^ Over 10 therapeutic monoclonal antibodies have been approved for use in immuno‐oncology, with targets that include B‐lymphocyte antigen (CD20), receptor tyrosine protein kinase erbB‐2 in breast cancers (HER2), vascular endothelial growth factor (VEGF), CD52, and CD33.^2^ New immunotherapies have also been successfully combined with existing therapeutic interventions. Co‐delivery of immunotherapy with chemotherapy, B‐Raf proto‐oncogene inhibitors, and VEGF‐directed therapy have all been shown to amplify antitumor responses.^3^ Newly developed virus‐like particles have demonstrated immunostimulatory capabilities which can be harnessed for immunotherapy for metastatic cancer.^4^ In addition, the field of T‐cell receptor engineering and the manufacturing of chimeric antigen receptor T‐cells (CAR T‐cells) have enabled improved immune recognition of tumor antigens.^5^

Despite this wealth of new technologies, the efficacy and widespread adoption of immunotherapy has been limited. The major challenge lies in delivering an immunotherapy to a specific target without causing harm to healthy tissues or inducing a feedback pathway that counteracts the mechanism of the immunotherapy. Nonspecific delivery of proinflammatory cytokines and monoclonal antibody therapies has the potential to induce systemic toxicity. In a similar fashion, the adoptive transfer of cells potentially can induce autoimmunity at off‐target sites.^6^ The development of cancer immunotherapies is stifled by the widespread presence of immune tolerance at the tumor site. Low immunogenicity of tumor antigens, the proliferation of immunosuppressive cells (e.g., myeloid‐derived suppressor cells, regulatory T‐cells), and the increased production of immunosuppressive cytokines (e.g., IL‐10, TGF‐β) work together to limit the antitumor response elicited by immunotherapies.^7^ Autoimmune diseases, conversely, have the opposite problem of inducing systemic immune suppression that renders patients susceptible to infectious disease.^8^ The overarching question is, therefore, how do we deliver the optimal amount of immunotherapy to a specific site, with appropriate kinetics and dosing schedule, without inducing deleterious side effects that outweigh the benefits of the therapy?

Nanoparticle platforms may serve as a solution to these drug delivery problems that constrain immunotherapy. Because of their larger size in comparison to small molecule therapeutics, nanoparticles have unique transport properties and biodistribution behavior. Moreover, the physical properties of nanoparticles (i.e., size, shape, charge, ligand density, and charge) can be engineered to facilitate the tuning of biodistribution, site‐specific targeting, immunogenicity, detectability by medical imaging, and therapeutic loading. Nanoparticles are capable of delivering immunomodulatory agents directly to the tumor microenvironment, inducing immune tolerance, and conjugating directly to adoptively transferred T‐cells for regulation of priming.^9^, ^10^, ^11^, ^12^ In addition, nanoparticles have been formulated to deliver cancer vaccines to antigen‐presenting cells. The enhanced delivery of antigens loaded onto nanoparticles as cancer vaccines is evident through decreased tumor proliferation in comparison to tumor treated with soluble antigen.^13^ When formulated as hydrophobic, solid‐in‐oil dispersions, nanoparticle delivery can be enhanced through the hydrophobic, protective stratum corneum of the epidermis. This enables transcutaneous vaccine delivery, which can be internalized by dendritic cells that subsequently traffic to the lymphatic system via the lymph nodes.^14^

Nanoparticle constructs also enable the intracellular delivery of DNA and RNA immunotherapeutics. Loading of nucleic acid therapeutics onto a nanoparticle significantly enhances its ability to travel to a target site and enter a cell. In addition to the widespread presence of RNA‐degrading enzymes in vivo, the delivery of free RNA molecules is impeded by their negative charge. This charge limits their ability to travel across the cellular membrane, which is also negatively charged.^15^ By the mechanism of silencing the production of inflammatory cytokines, nanoparticles loaded with siRNA have demonstrated potency against melanoma. Therapeutic efficacy is even further enhanced when the siRNA nanoparticles are delivered concurrently with nanoparticles that deliver tumor antigen and immune adjuvants, such as the CpG oligonucleotide.^16^, ^17^ Concurrent delivery of pDNA antigen, CpG oligonucleotide, and siRNA targeted to IL‐10 was able to enhance the Th1/Th2 cytokine ratio to favor an antitumor response.^18^ Another nanoparticle vaccine consisting of an immune response modifier, imiquimod, and STAT3 siRNA boosts the expression of co‐stimulatory molecules (CD86), increases the production of IL‐2, and enhances cytolytic T‐cell activity after delivery to dendritic cells.^19^ To present a wider array of antigens to boost the antitumor response, tumor lysate vaccines have been developed. It appears to be advantageous to deliver tumor lysate on nanoparticles; lysate‐loaded particles were able to stimulate dendritic cell migration, upregulate co‐stimulatory and MHC expression, and slow tumor growth to a greater degree than tumor lysate in soluble form.^20^ It should be noted that combinatorial nanoparticle therapies are not limited to the delivery of vaccines. Polyamine/lipid nanoparticles loaded with siRNA designed for several gene targets were designed for delivery to the vascular endothelium. The construct was able to effectively simultaneously silence Tie1, Tie2, VEGFR‐2, VE‐cadherin, and ICAM‐2 specifically in lung endothelial cells in vivo.^21^ Depending on the material property of the selected nanoparticle, biodistribution can also be monitored using medical imaging modalities (i.e., iron oxide nanoparticles for magnetic resonance imaging applications). This strategy was applied to monitor the efficacy of gene therapy to mitigate immune rejection of heart transplants in rats.^22^

The versatility of nanoparticles suggests that they can easily elevate immunotherapy efficacy to another level, but in reality, their efficacy is limited by a set of unique drug delivery problems. Nanoparticle targeting may be slightly more specific than small molecule targeting, but serum protein opsonization usually leads to their accumulation in phagocytic cells. In addition, there are some tissue interfaces (e.g., the blood brain barrier) which are not conducive to nanoparticle penetration.^23^ Nanoparticles do have the enhanced ability to accumulate by passive targeting into highly angiogenic tumors. Increased vascular permeability, which is due to rapid tumor angiogenesis, permits extravasation of nanoparticles into the tumor interstitium by a phenomenon known as the “Enhanced Permeation and Retention Effect.” High interstitial pressures caused by the extravasation of proteins that stifle lymphatic flow, however, often impede the flow of nanoparticles into the tissue.^24^ If a nanoparticle can successfully evade phagocytic clearance, it then faces the challenge of traveling to its targeted cellular compartment for its intended biological effect to be realized. For example, the delivery of immune adjuvants is often targeted to specific TLRs, retinoic acid‐inducible gene 1 (RIG‐I) like receptors, or nucleotide‐binding oligomerization domain (NOD)‐like receptors, which may be located on the cell membrane, on membrane‐bound organelles, or in the cytoplasm.^25^, ^26^ The delivery of DNA and interfering RNA requires localization to the nucleus or the cytoplasm, respectively.^27^ Unfortunately, nanoparticles have a tendency to traffic through vesicles by the clathrin‐mediated endocytosis, caveolae‐dependent endocytosis, or micropinocytosis pathway. All of these pathways converge into the endolysosomes, where low pH deactivates nucleic acids.^28^ Further engineering is required to deliver a RNA‐loaded nanoparticle to the cytosol, where interaction with the RNA interference silencing complex can occur.

**Figure 1:**
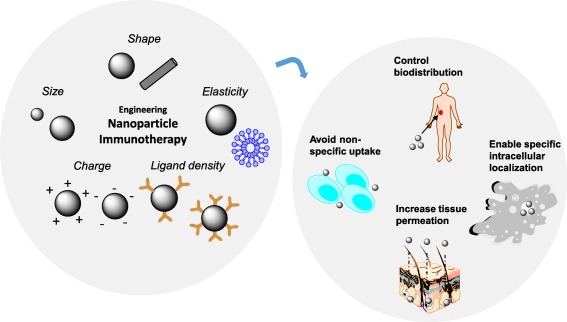
Engineering nanoparticle immunotherapy. Nanoparticles of unique size, shape, elasticity, charge, and ligand density can be formulated to enhance the delivery of immunotherapies. Through an understanding of the effect of each of these parameters on biotransport and immunogenicity, nanoparticles can be designed to control the biodistribution of immunotherapies, evade nonspecific uptake by phagocytic cells, increase tissue permeation, and enable specific localization to targeted cellular compartments

In this review, we will evaluate how nanoparticles can be engineered so they can overcome these obstacles and deliver immunotherapies more efficiently to their target sites (Figure 1). First, we will discuss how the size, shape, and surface chemistry of a nanoparticle affects multiple biological processes. We will focus on how these nanoparticle design parameters influence cellular recognition and internalization, transport through the vasculature, biodistribution, and the elicited immune response. Then, we will discuss methods in which nanoparticles can be engineered to maximize immunotherapeutic efficacy. The engineering approaches discussed will include (a) targeting immunotherapeutic nanoparticles to specific tissues and cells, (b) using environment‐sensitive biomaterials to optimize immunotherapy delivery from nanoparticles, (c) designing nanoparticles that are able to penetrate into deep tissue, (d) optimizing the intracellular delivery of nanoparticles for gene delivery and RNA interference, (e) designing nanoparticles to enhance vaccine delivery, and (f) designing nanoparticles to boost the antitumor immune response (Table 1).

**Table 1:** Strategies to enhance the efficacy of nanoparticle immunotherapy

Strategy	Nanoparticle design implementation	References
Reducing nonspecific uptake	Hydrophilic polymers (e.g., PEG)	29
	Engineering of shape	30
	Cellular hitchhiking	31, 32
	Engineering of nanoparticle rigidity	33
Enhancing tissue permeation	Tissue‐penetrating peptides	34, 35, 36, 37, 38, 39
	Chemical modification to increase permeation	40, 41, 42
Targeting to immune cells	Dendritic cell and macrophage targeting	43, 44
	T lymphocyte targeting	45, 46
	B lymphocyte targeting	47, 48
Targeted intracellular delivery	Cationic polymers	49, 50
	pH‐sensitive biomaterials	51, 52, 53
	Virus‐derived cell‐penetrating peptides	54, 55, 56
	Direct cytosolic delivery via scavenger receptor	57, 58
Controlling release kinetics	Controlled release rate	59, 60
	Enzyme‐triggered release	61, 62
	Photothermal triggered release	63, 64, 65
**Application**	**Nanoparticle design implementation**	**References**
Boosting nanoparticle vaccines	Manipulating antigen presentation	66, 67, 68, 69
	Lymphoid organ targeting	70, 71, 72, 73, 74, 75
Boosting the anti‐tumor immune response	Stimulating immune activation	76, 77, 78, 79
	Targeting the tumor microenvironment	80, 81, 82, 83

## 2 Engineering Nanoparticles to Manipulate Transport and the Immune Response

The physical characteristics of a nanoparticle critically affect its in vivo transport and the immune response it triggers. Each design parameter has its own unique contributions to an immunotherapeutic nanoparticle's biostransport and toxicity profile. We highlight the effect of manipulating the design parameters of nanoparticle size, shape, charge, ligand density, and elasticity individually, but all parameters and their interplay must be considered when formulating a nanoparticle with efficient therapeutic delivery and low toxicity.

### 2.1 Nanoparticle size

Nanoparticle size is a design parameter that can be tuned to enhance the targeted delivery and subsequent efficacy of nanoparticle immunotherapies. The size of a nanoparticle critically affects its pharmacokinetics, vascular transport, and cellular uptake. While small nanoparticles (<10 nm) have a tendency to be cleared in the kidneys, larger nanoparticles are more likely to be cleared by the liver and the spleen.^84^ Nanoparticles that are greater than 200 nm in diameter are similar in size to fenestrations in the spleen, so particle elasticity also plays a role in the rate of splenic clearance for larger particles.^85^ In addition to affecting the clearance rate, nanoparticle size affects how the particle's transport is mediated by blood flow. While the motion of smaller nanoparticles is primarily governed by diffusion, the motion of larger nanoparticles is governed by a combination of diffusion and convective flow.^86^ The size effect on nanoparticle transport, therefore, has ramifications on how efficiently a nanoparticle can extravasate from a blood vessel and enter either a tumor or an inflammation site. When interstitial pressures are high, fast blood flow is required to direct larger nanoparticles deep into the tumor interstitial space. Therefore, large nanoparticles have a variable intratumoral distribution which depends on regional blood flow.^87^ Whole body biodistribution of nanoparticles is also influenced by particle size. In a study comparing polystyrene spheres of diameters ranging from 0.1 to 10 μm, particle accumulation in the liver decreased as particle size increased. At the same time, particle accumulation in the lungs increased as particle size increased.^88^ This difference in uptake behavior highlights that the optimal particle size for cellular internalization is dependent on cell type. Moreover, size may be optimized to maximize the rate of uptake into cells. With HeLa cells, it was observed that the uptake of 50 nm nanoparticles was increased over the uptake of smaller 14 nm nanoparticles and larger 74 nm nanoparticles.^89^ The effect of nanoparticle size on pharmacokinetics, transport, and internalization will manifest itself in downstream effects as well. To evaluate the effect of size on nanoparticle immunogenicity, micro‐ and nano‐sized polylactide particles were formulated and loaded with pneumococcal antigens. It was subsequently found that the IgG responses to the particle vaccine depend on size.^90^ An evaluation of nanoparticles manufactured by the Particle Replication in Nonwetting Templates (PRINT) technology, loaded with the ovalbumin (OVA) antigen, showed that 80 × 180 nm PEGylated nanoparticles elevated anti‐OVA IgG titers significantly more than 1‐µm PEGylated nanoparticles. Moreover, size is dominant over polyethylene glycol (PEG) linker length in influencing the humoral response.^91^

### 2.2 Nanoparticle shape

**Figure 2:**
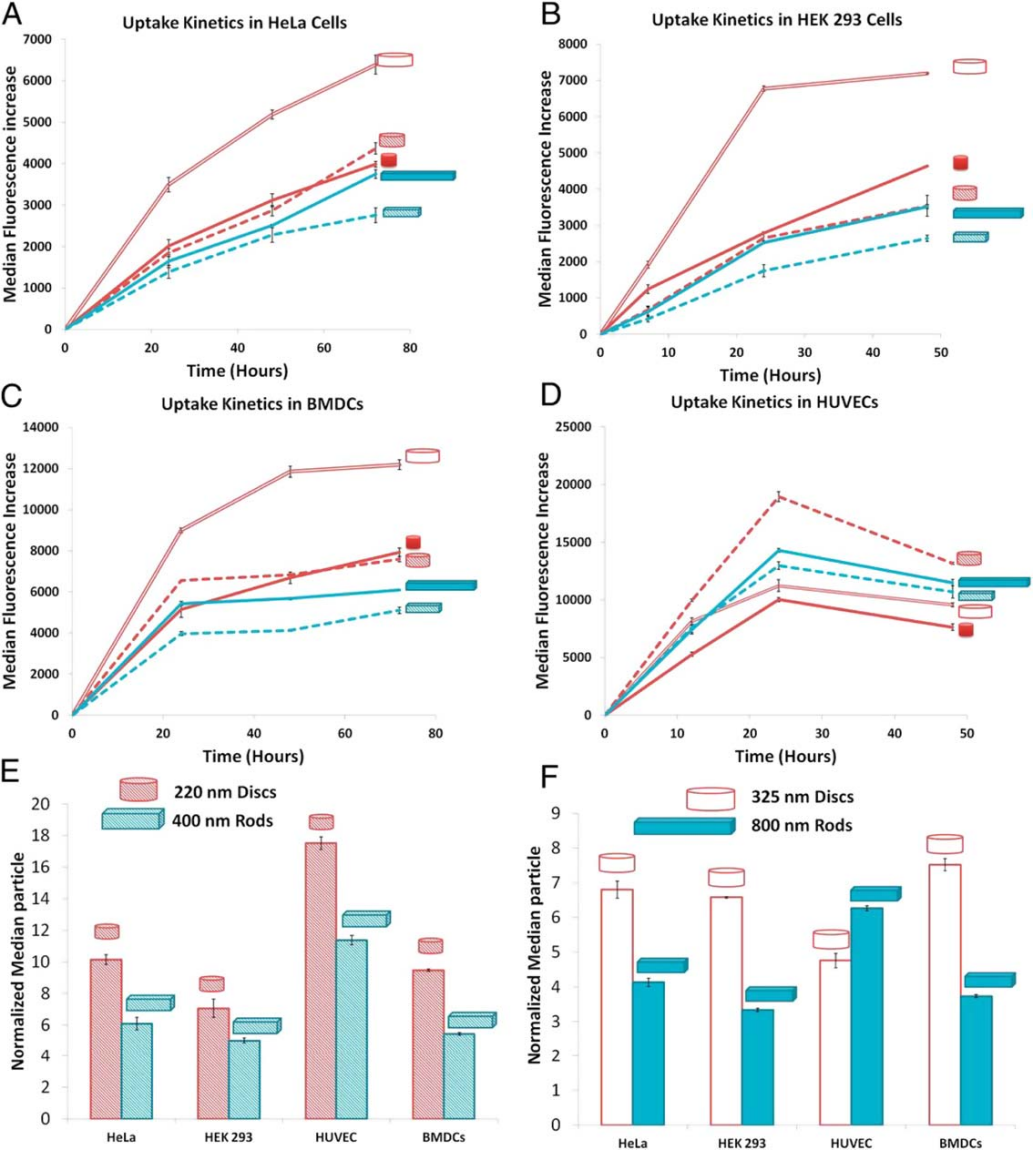
Shape affects the internalization of nanoparticles. Cellular‐uptake kinetics of different shape‐specific nanoparticles in various cell lines. (A) HeLa cells, (B) HEK 293 cells, (C) BMDCs, and (D) HUVEC cells. In A–D, red lines are for nanodiscs (hollow for 325 × 100‐nm disks, dashed for 220 × 100‐nm disks, and solid for 80 × 70‐nm disks), and blue lines are for nanorods (dashed for 400 × 100 × 100‐nm rods and solid for 800 × 100 × 100‐nm rods). Error bars are *SD* with *n* = 3 for each data point. (E, F) Normalized median particle uptake per cell (indicates relative number of particles internalized by cells when normalized to 100 particles of 80 × 70‐nm disks) at the maximum internalization time point (72 hr for HeLa and BMDC, 48 hr for HEKs, and 24 hr for endothelial cells). Reproduced with permission from ref. 95

Particle shape is also a critical design parameter that influences how a nanoparticle immunotherapy moves while in the blood circulation, becomes internalized by cells, and stimulates an immune response. Initial nanoparticle formulations were produced primarily in spherical shapes, but recent advances in nanoparticle engineering have generated a wide portfolio of shapes that include rods, prisms, cubes, stars, and disks.^92^ Nanoparticle asymmetry promotes particle tumbling toward the wall of blood vessels under flow (margination), which is caused by a nonuniform distribution of hydrodynamic forces acting on the particle. Asymmetry of nanoparticles also enhances nanoparticle penetration and distribution inside solid tissues and tumors. Inside tumor spheroids, nanodisks were observed to accumulate at higher amounts throughout a tumor spheroid than similarly sized nanorods. This difference in accumulation can be explained by the difference in interactions that particles of different shapes have with the cell membrane. As asymmetric nanoparticles approach the cell membrane at different contact angles, their rates of internalization will differ.^93^ More specifically, this interaction angle will affect the energy required for particle internalization.^94^ When an elongated particle's major axis lies tangential to a cell membrane, internalization is more difficult than if the particle's minor axis was aligned tangential to the cell membrane. In agreement with these findings, hydrogel nanorods with a higher aspect ratio were internalized by cancer cells, endothelial cells, and dendritic cells more slowly than hydrogel nanodiscs. This difference in uptake rate could be attributed to differences in adhesion forces between the cell membrane and particles of different shapes, the strain energy required for membrane deformation, and the effect of sedimentation or local particle concentration at the cell surface (Figure 2).^95^

Like size, a nanoparticle's shape also influences the immunological response to the particle. An evaluation of spheres, cubes, and rods designed to stimulate antibody production against the West Nile Virus demonstrated that spheres and cubes induce the secretion of proinflammatory cytokines (TNF‐α IL‐6, IL‐12, GM‐CSF), while rods induce the secretion of inflammasome‐related cytokines (IL‐1β, IL‐18). Interestingly, the spheres and cubes were concurrently not internalized efficiently as rod‐shaped particles. The aspect ratio of a rod‐shaped particle also influences dendritic cell maturation and production; rods with a high aspect ratio induce significantly higher production of IL‐1β, IL‐6, and IL‐12 than rods with a shorter aspect ratio.^96^ An evaluation of both nanoparticle size and shape suggest that surface area is a key parameter that influences the immune response.^97^ This assertion is backed by the observation that cytokine production (TNF‐ α, IL‐6) was also induced to different degrees by triangular, square, and pentagonal RNA nanoparticles in mouse macrophage cultures.^98^ It has also been suggested that particle shape influences the Th1/Th2 polarization of the immune response. In a comparison of spherical and rod‐shaped nanoparticles, it was found that spheres produced a Th1 biased response against OVA, while rods produced a Th2 biased response.^99^ It is, therefore, important to consider that nanoparticle shapes may trigger different intracellular signaling pathways, which can lead to unique immune responses.

### 2.3 Nanoparticle charge

The tuning of nanoparticle surface chemistry has enabled a breadth of applications for nanoparticle immunotherapeutic delivery. Introduction of charge to nanoparticles enables the loading of moieties on the particle surface by electrostatic interactions. Linear polyethylenimine (lPEI) has been established as a cationic polymer which can complex with DNA and RNA and deliver its cargo into the cytoplasm.^100^ PEI can be further modified to include degradable linkers and functional groups to enhance cellular uptake.^101^ Through similar mechanisms, cationic lipid nanoparticles also can shuttle siRNA into the cytoplasm of cells.^102^ The surface charge of a nanoparticle has the dual effect of enabling gene delivery and modulating the immune response. When positively charged, antigen‐conjugated nanoparticles for pulmonary immunization stimulate antibody production, germinal B‐cell expansion, CD4+ T‐cell activation, and expression of MHC II and coactivating receptors on T‐cells.^103^ When charge and hydrophilicity are varied in mesoporous silicon nanoparticles, downstream CD3, CD4, and CD8 proliferation are altered and leads to biasing toward a Th1 or Th2 response.^104^ Application of positively charged or negatively charged nanoparticles to the skin revealed that positive charge could deepen skin penetration 2–6 fold.^105^

A major issue with cationic nanoparticles has been acute systemic toxicity and nonspecific systemic immune‐stimulation. It is widely accepted that almost all cationic particles stimulate acute inflammation, the exact mechanism of which are still being elucidated. A potential mechanism could be that cationically charged polymers used to synthesize nanoparticles can trigger pattern‐recognition receptors in immune cells.^106^ Several reports have indicated that the polysaccharide chitosan induces inflammasome response.^107^, ^108^ This toxicity/immunogenicity issue hinders nanoparticle usage in the clinic and many promising formulations that showed encouraging results in vitro have failed to move past preclinical studies. Significant effort must be invested in understanding how charged nanoparticles (as well as uncharged ones) interact with the serum and extracellular molecules once injected and how the so called “corona effect” as well as the fundamental structure of the nanoparticle‐material induces specific signaling pathways in immune cells, endothelial cells (which could be a major source of proinflammatory cytokines), and fibroblasts.^109^

### 2.4 Nanoparticle ligand density

In addition to size, shape, and charge, the ligand density of a nanoparticle plays a critical role in its biodistribution, cellular uptake, cellular association, and immune response. Ligand conjugation to a nanoparticle can reduce off‐target cytotoxicity. Simple functionalization of mesoporous silicon nanoparticles with amines can significantly reduce systemic toxicity and increases the maximum tolerated dose of particles.^110^ In addition, polymers such as PEG have long been used to enhance particle hydrophilicity and reduce clearance by the reticuloendothelial system. Studies evaluating the effect of PEG density have also revealed that the degree of PEG surface loading changes the particle biodistribution between hepatocytes and Kupffer cells.^111^ In addition to mediating the rate of off‐target particle uptake, ligand density and distribution also influences the rate of internalization into targeted cells.^112^ Internalization and externalization rates of a targeted receptor will affect the optimal ligand density that maximizes nanoparticle uptake, as was seen with folate‐targeted liposomes.^113^ Particle shape must also be taken into consideration when determining optimal ligand density. For a ligand with a fixed length and flexibility, a spherical nanoparticle will interact with a different number of receptors at an interface than an asymmetric nanoparticle. This was observed in a study comparing the association of symmetric and asymmetric ligand‐decorated PRINT hydrogels with alveolar macrophages.^114^ Not surprisingly, enhancement in the cell uptake of nanoparticles correlates to enhancement in therapeutic efficacy due to the nanoparticle. When the antibody surface density was increased on DEC‐205 targeted nanoparticle dendritic cell vaccines, the rate of uptake and subsequent induction of CD36 expression on dendritic cells increased.^115^ Ligand spacing and multiplicity have also been demonstrated to critically affect peptide‐DNA complex induced TLR9 activation.^116^

### 2.5 Nanoparticle elasticity

Tuning the flexibility of a nanoparticle also affects antibody‐mediated targeting, endocytosis, and phagocytosis. Softer particles have a prolonged blood circulation residence time and increased organ deposition. It is hypothesized that this is caused by the deformation of softer particles by macrophages into shapes which are more difficult to internalize.^117^ Particle endocytosis, however, occurs more quickly with flexible nanoparticles. When the rates of endocytosis of HER2 targeted flexible liposomes and rigid mesoporous silica particles into HT29 colon cancer and SKBR3 cancer cells were compared, it was found that the liposomes underwent endocytosis more quickly than the silica particles.^118^ These findings reflect the importance of considering particle design, cell type, and the mode of internalization when developing a nanoparticle immunotherapy for a given application.

## 3 Improving Nanoparticle Design to Enhance Immunotherapy Efficacy

### 3.1 Designing nanoparticles to control where and when immunotherapies are delivered

#### 3.1.1 Reduction of nonspecific uptake

Designing a nanoparticle that avoids nonspecific uptake is as important as designing a nanoparticle which can be internalized efficiently by its target. Hydrophilic polymers, such as PEG, have long been used in nanoparticle formulations to reduce macrophage uptake and promote long circulation. Anchoring of anti‐CD40 antibodies and CpG oligonucleotides to PEGylated lipid nanoparticles decreases the incidence of off‐target side effects while maintaining therapeutic efficacy.^29^ Engineering of nanoparticle shape will also aid nanoparticles to evade the macrophages of the reticuloendothelial system. For example, shaping mesoporous silica nanoparticles as long rods will slow their rate of excretion when compared to mesoporous silica nanoparticles shaped as short rods.^30^ Another means to hide from circulating macrophages is a process known as “cellular hitchhiking,” in which nanoparticles act as stowaways on the surface of nonimmunogenic cells. The cloaking of nanoparticles with the cell membranes of red blood cells (RBCs) has proven useful in Type II autoimmune diseases, where antibodies opsonize RBCs for phagocytosis. Cell membrane decorated nanoparticles can also serve as a sink for anti‐RBC antibodies, which prevents phagocytosis and subsequent destruction of healthy RBCs by macrophages.^31^ The incorporation of cell membrane components to polymeric poly(lactic‐co‐glycolic acid) (PLGA) particles provides the added benefit of the ability to load lipid adjuvants (e.g., monophosphoryl lipid A), to enhance the efficacy of tumor vaccines.^119^ There has also been implementation of an elegant strategy that reduces nonspecific nanoparticle uptake through combination of all of the aforementioned approaches: active targeting, shape optimization, and cellular hitchhiking. Combination of these three approaches was able to significantly reduce off‐target accumulation of nanoparticles targeted to ICAM‐2 on the vascular endothelium in the lung.^32^ Nanoparticle rigidity may be another parameter that can be altered to reduce nonspecific nanoparticle uptake. In a comparison of soft and rigid discoidal polymeric nanoparticles, it was found that softer nanoparticles were internalized by macrophages less quickly than their rigid counterparts.^33^

#### 3.1.2 Enhancing tissue penetration

**Figure 3:**
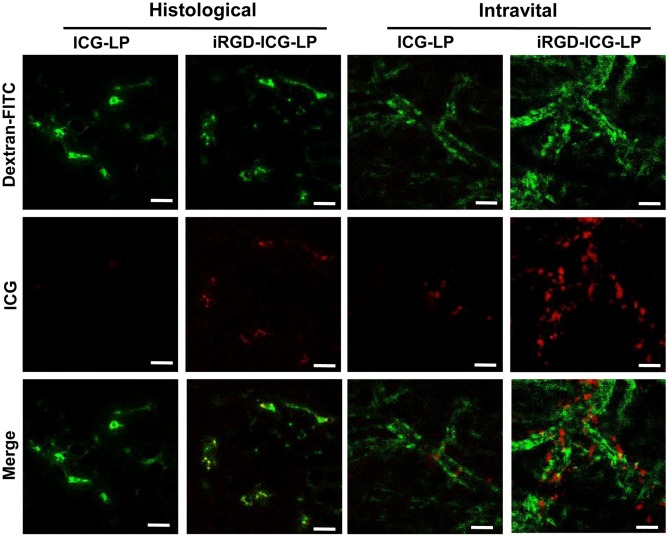
iRGD enhances the endothelial permeation of indocyanine‐labeled liposomes. The binding and penetration of iRGD–ICG‐LPs or ICG‐LPs to angiogenic endothelial cells were assessed with intravital and histological examination. The tumor vascular images were captured at 10 min after injecting 40 kDa FITC‐Dextran. The frozen sections were examined under a confocal microscope. Green represented the blood vessels labeled by FITC‐Dextran and red represented ICG‐loaded nanoparticles (Scale bar, 50 μm). Reproduced with permission from ref. 37

As with nanoparticle chemotherapy, it is challenging to deliver nanoparticle immunotherapy deep into a tumor. The rapid rate of tumor development results in the generation of highly vascularized regions at the periphery of a tumor surrounding an avascular core.^120^ Blockage of the lymphatic system with extravasated proteins causes high interstitial pressures, which also hinder the transport of nanoparticles into the tumor site. Therefore, it is a significant challenge to increase the distance in which nanoparticles travel from a blood vessel into the deep tissue space. To overcome this challenge, peptide and chemical modifications to the nanoparticle surface have been developed to improve the tissue penetration.^34^ For example, polyarginines have been used to enhance the skin permeation of lipid nanoparticles. An added benefit of surface functionalization with polyarginines is that the modified particles had increased retention in the dermis after administration.^35^ Another such “tumor‐penetrating peptide” is cyclic CRGDK/RGPD/EC, cyclized between the two cysteines with a disulfide bond iRGD, which binds to overexpressed αv integrins. After binding to αv integrin, a proteolysis‐induced structural change converts iRGD to a substrate for neuropilin‐1 and neuropilin‐2. Proteins of the neuropilin family facilitate angiogenesis after interaction with VEGF, of which overexpression is frequently a hallmark of tumor progression. Through this mechanism, the iRGD peptide is able to deliver coadministered dextran into peritoneal tumors and increase the therapeutic index of the chemotherapeutic doxorubicin.^36^ Administration of iRGD‐conjugated indocyanine liposomes to angiogenic endothelial cells also confirmed that iRGD enables nanoparticle permeation beyond the vascular endothelium (Figure 3).^37^ When iRGD was conjugated to a microenvironment‐responsive and multistage nanoparticle and administered to mice with 4T1 orthotopic breast tumors, increased nanoparticle permeation was observed throughout the tumor and was accompanied by a decrease in tumor burden.^38^ Tumor‐penetrating and membrane‐translocating peptides have also been used to enhance the transport of nanocomplexes with siRNA to silence ID4, a prominent oncogene, in ovarian tumors. Conjugation of a tumor‐penetrating peptide to the nanoparticle enabled deep localization away from the vascular endothelium and significantly enhanced siRNA delivery in comparison to naked nanoparticles.^39^ Addition of these peptides to nanoparticle immunotherapies can potentially equalize distribution within both tissues and tumors. Cell‐penetrating peptides have already been evaluated to enhance the delivery of vaccines to the mucosa. In an intranasal application, poly(N‐vinylacetamide‐*co*‐acrylic acid) was modified with d‐octaarginine to deliver OVA to the mucosa. The cell‐penetrating peptide modified vaccine significantly elevated OVA‐specific IgG titers in the sera of vaccinated mice.^121^

Chemical modification of nanoparticles can also modify its permeation and cell‐penetration properties. For instance, the addition of Pluronic F127 modified lipid vesicles to chitosan nanoparticles was found to enhance epithelial mucosa penetration. Furthermore, the addition of a polyethylene oxide corona could further improve transport of the nanoparticles throughout the mucosa.^40^ Imidazole modification has also been shown to increase the tissue and mucosa permeation of chitosan nanoparticles, which provides an opportunity for the delivery of DNA vaccines and immunotherapeutic siRNA delivery.^41^ Intravenous administration of imidazole‐modified chitosan‐siRNA nanoparticles led to a 49% knockdown in GAPDH protein expression in the lungs, while unmodified nanoparticles only induced 11% knockdown.^42^

#### 3.1.3 Targeting nanoparticles to immune cells

As mentioned, one challenge that limits the efficacy of nanoparticle immunotherapy is determining how to control nanoparticle biodistribution. One common strategy to enhance nanoparticle immunotherapy is to attach a ligand or peptide that facilitates homing to a particular target. Monoclonal antibodies can also be conjugated to nanoparticles for the specific delivery of antigens and adjuvants to dendritic cells (anti‐CD40) for tumor vaccination and TGF‐β and IL‐2 to induce regulatory T‐cells (anti‐CD4) for the downregulation of autoimmune disease.^43^, ^44^ Nanoparticles targeted to dendritic cells using monoclonal antibodies against CD40, DEC‐205 (C‐type lectin), and CD11c (integrin receptor) were able to stimulate IL‐12 production and co‐stimulatory marker CD86 more effectively than nontargeted nanoparticles.^122^ Nanoparticles for immunotherapy can also be targeted to dendritic cells and macrophages through functionalization with mannose, which binds to the mannose receptor (CD206). Mannose conjugation to nanoparticles coloaded with tumor vaccines and TLR adjuvants elevated interferon‐γ levels in the spleen and slowed tumor proliferation in comparison to untargeted nanoparticles.^45^ The success of this approach is not limited to mannose; a galactosylated cationic dextran has also been used to formulate nanoparticles that can deliver oligonucleotides to tumor‐associated macrophages 2.5 times more efficiently than their ungalactosylated counterparts.^46^

Cell targeting of nanoparticles is not limited to antigen presenting cells—methods have been also developed to target nanoparticles to B and T lymphocytes for immunotherapeutic delivery. Antibody fragments specific for cell surface antigens on adoptively transferred T‐cells have been used for the targeted delivery of liposomes with IL‐2.^123^ Dendrimers, which have the advantage of multivalent targeting capability, have been used to deliver small molecule payloads to B‐cells.^47^ Calcium phosphate nanoparticles loaded with protein antigen have also been developed to activate antigen‐specific B‐cells. The antigen‐specific B‐cells could internalize a large number of the nanoparticles, which resulted in increased expression of the early activation marker CD69, the increased expression of co‐stimulatory marker CD86, and extensive cross‐linking of the B‐cell receptors.^48^

#### 3.1.4 Tailoring the intracellular delivery of nanoparticle immunotherapies

Once a nanoparticle immunotherapy reaches its target cell, it must be able to travel to the appropriate intracellular compartment for biological activity to occur. For the delivery of DNA plasmid vaccines, trafficking to the nucleus is required. For the application of delivering siRNA to inhibit checkpoint blockade, nanoparticles must be able to escape the endosomal pathway into the cytoplasm, where interaction with the RNA‐induced silencing complex occurs. Initial attempts at facilitating the endosomal escape of nanoparticles involved polymeric modifications to create a cationic particle surface (e.g., polyethylenimine).^49^ It has been proposed that endosomal escape is then induced by a “proton sponge effect.” The effect is due to the protonation of cationic polymers at low pH, which causes an influx of protons followed by an influx of chloride ions and water. Rapid water influx is then hypothesized to cause the lysosome to swell and eventually burst, which frees nanoparticles from the endolysosomal trafficking pathway.^50^ More advanced strategies to facilitate nanoparticle escape into the cytosol rely on the lower pH in the milieu of the late endosomes and lysosomes. One antigen delivery system employs an antigen‐loaded liposome modified with biodegradable polysaccharides that become fusogenic under acidic conditions. After fusion with the endosomal membrane, the particle can deliver antigens into the cytoplasm. These antigens can subsequently be presented to CD8+ T‐cells and induce cytotoxic T‐lymphocytes.^51^ In pH sensitive galactosyl dextran‐retinal nanogels, it is hypothesized that the cleavage of hydrazone bonds at acidic pH both disassemble the nanogel and induce lysosomal rupture by the proton sponge effect.^52^ Micelleplexes that disrupt the lysosomal membrane by two separate mechanisms have also been developed. The cationic micelleplex can become protonated at endosomal pH, which induces the proton sponge effect. In addition, the pH sensitive micelleplex was designed to release amphotericin that creates pores which further destabilize the endolysosomal membrane.^53^

**Figure 4:**
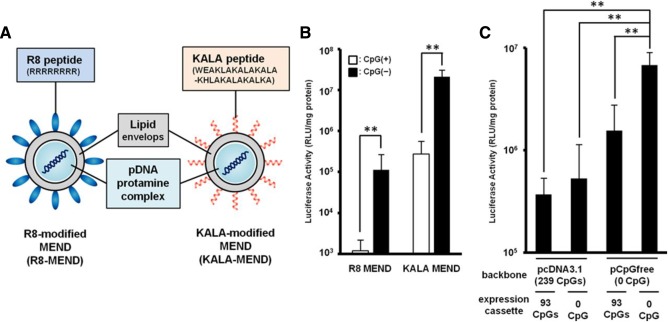
Viral peptides enhance the endosomal escape of nanoparticles. (A) Illustration of the R8‐MEND (left) and KALA‐MEND (right). (B) The R8‐MEND and KALA‐MEND encapsulating a conventional pDNA (pcDNA3.1‐Luc; opened bar) or CpG‐free pDNA (pCpGfree‐Luc(0); closed bar) were transfected to BMDCs. Data were presented as the mean ± *SD* of three independent experiments. Statistical differences were evaluated by one‐way ANOVA, followed by Student's *t* test (**p < .01). (C) The transfection activity of KALA‐MENDs encapsulating a pDNA with various set of backbone and inserts was also evaluated. Data were presented as the mean ± *SD* of three independent experiments. Statistical analyses were performed by one‐way ANOVA, followed by Bonferroni test. **p < .01 versus pCpGfree‐Luc(0). Reproduced with permission from ref. 56

Another means of enhancing the intracellular delivery of nanoparticle immunotherapies is to incorporate cell‐penetrating peptides on the particle surface. When attached to small molecules, cell‐penetrating peptides can translocate with their cargo across the plasma membrane. Nanoparticles with cell‐penetrating peptides conjugated to their surface will continue to enter cells through endocytic pathways; the peptides, however, enable the nanoparticle to penetrate the membrane of the endolysosomes to gain entry into the cytosol.^54^ More advanced cell‐penetrating peptides are derived from viral coat proteins, which enable viruses to escape the endosome and proceed to infect its host. Viral pH sensitive peptides, such as GALA or KALA, undergo a conformational change at low pH that promotes the destabilization and fusion of lipid membranes. It has been validated that these pH‐sensitive, fusogenic peptides can be conjugated to cationic liposomes to boost transfection efficiency.^55^ Conjugation of KALA to lipid nanoparticles also have demonstrated increased immunostimulatory abilities, measured by the upregulation of interferon‐γ, IP‐10, and IL‐1β from bone marrow‐derived dendritic cells, when compared to soluble CpG or nanoparticles without KALA (Figure 4).^56^ GALA‐modified lipid nanoparticles have also been used to deliver siRNA targeting SOCS1 (suppressor of cytokine signaling) in dendritic cells, which led to enhanced phosphorylation of STAT1 and the increased production of proinflammatory cytokines.^124^ More elaborate nanoparticle designs enable targeted mitochondrial delivery through a mitochondria‐fusogenic lipid envelope surrounded by an endosome‐fusogenic lipid envelope. The exterior lipid envelope facilitates endosomal escape and a mitochondrial targeting signal enable fusion of the nanoparticle with the mitochondrial membrane, where cargo can be delivered.^125^

Yet, even another strategy to enhance intracellular delivery is to bypass traditional mechanisms of nanoparticle internalization (clathrin‐mediated endocytosis, caveolae mediated endocytosis, micropinocytosis) altogether. These alternative internalization routes fully bypass the endolysosomal pathway without disrupting the intracellular vesicular compartments. One target pathway is the scavenger receptor BI (SR‐BI), which can be targeted with high density lipoproteins (HDLs).^57^ Reconstituted HDL has been combined with cholesterol‐siRNA to make siRNA nanocarriers that can bypass the endolysosomal pathway and reach the cytosol, where silencing can occur. The HDL‐modified cholesterol‐siRNA carriers could successfully downregulate VEGF expression in a MCF‐7 breast tumor model that expressed the SR‐BI receptor.^58^

With all of these intracellular delivery strategies, it is important to consider the balance between efficient delivery and toxicity and immunogenicity. Cationic polymers, while effective at facilitating endosomal escape, also may induce cytotoxicity at high doses. Traditional cell penetrating peptides, which permeate the cell membrane, also will cause harm to cells at very high doses.^126^ If new fusogenic viral peptides are conjugated to a nanoparticle, it is essential to evaluate if their systemic immunogenicity are outweighed by the increased therapeutic efficacy which is enabled by more site‐specific intracellular delivery.

#### 3.1.5 Controlling release kinetics of nanoparticle‐based immunotherapies

So far, we have discussed ways to deliver nanoparticles to targeted locations and also ways to prevent delivery to undesired locations. In addition to where an immunotherapy is delivered, it is also important to consider when and for how long it is delivered. The intelligent design of nanoparticles for immunotherapy delivery can facilitate such precise control of release kinetics. This is especially important in immunotherapy applications for autoimmune disease, which requires prolonged maintenance of therapy. One approach to control kinetics is to use degradable nanoparticles, which can deliver immunotherapies with higher efficacy, at a slower rate, and with reduced toxicity. Biodegradable PLGA nanoparticles can deliver TLR adjuvants over several days, which have the advantage of increasing adjuvant uptake by dendritic cells and prolonging dendritic cell activation.^59^ When nanoparticles are used to slowly deliver antigen over a long period of time, it is evident that the long‐term memory response is enhanced.^60^

Another approach to control when an immunotherapy is delivered is to incorporate functionalities that trigger drug release by internal or external mechanisms. We have already mentioned therapeutic approaches that rely on pH changes to facilitate site‐specific intracellular delivery. The use of enzyme‐sensitive linkers could also be incorporated into future nanoparticle designs for controlled release. For example, the incorporation of a matrix metalloprotease‐2 responsive linker into a nanoparticle could facilitate the specific delivery of an immunotherapy in the tumor microenvironment.^61^ In a similar fashion, incorporation of matrix metalloproteinase‐9 sensitive lipopeptides into liposomes may facilitate the release of immunotherapy specifically at the location of a tumor.^62^ External mechanisms to trigger immunotherapy release primarily rely on the use of near‐infrared light to trigger chemical degradation or thermal ablation. For example, triggerable copper sulfide (CuS) nanoparticles have been formulated with a chitosan surface coating to deliver CpG oligonucleotide. Upon excitation with near‐infrared light, the nanoparticles undergo disintegration and reassemble into polymer complexes exhibiting enhanced tumor retention. In addition, the new complexes can traffic efficiently to TLR‐9‐expressing‐endosomes in plasmacytoid dendritic cells, which promotes innate immunity through the activation of natural killer cells. At the same time, photothermal ablation can perturb the tumor microenvironment and dislodge tumor antigens for recognition and ingestion by antigen presenting cells within the tumor stroma. The subsequent activation of antigen presenting cells will lead to cross‐priming of tumor antigen‐specific T‐cells within the tumor draining lymph nodes.^63^ Gold nanoparticle photothermal therapy has also been used for immunomodulation in tumors. It has been observed that photothermal therapy can promote a tumor‐specific immune response in melanomas, which resulted in extensive proliferation of CD4+ helper T‐cells and CD8+ cytotoxic T‐cells.^64^ Thermal ablation facilitated by gold nanoparticles also generates danger‐associated molecular patterns, which activate inflammasome complexes that activates caspases to cleave precursors of proinflammatory cytokines.^65^

### 3.2 Designing nanoparticles to fine‐tune the immune response in vaccines and tumor immunotherapies

#### 3.2.1 Nanoparticle vaccines

Nanoparticle vaccines have been established to significantly upregulate T‐cell responses over equivalent soluble vaccines.^66^ To maximize this effect, it is essential to consider both a nanoparticle vaccine's physical characteristics and material to induce a specific, desired immune response. For instance, the immune response elicited from a polymersome, which is a watery core particle, differs from the immune response elicited by a solid core nanoparticles composed of poly(propylene sulfide) (PPS). The polymersome encapsulates its antigen cargo, while antigen is conjugated to the surface of the PPS nanoparticle. It was found that antigen delivered in polymersomes tended to enhance CD4 responses, while antigen delivered by PPS nanoparticles tended to enhance CD8 responses. These differences are attributed to the difference in tendency to present antigen to MHC II or MHC I pathways.^67^ Nanocarrier porosity also affects the manner in which antigen is encapsulated and how downstream immune responses are elicited. Three silica nanocarriers of different porosities each induced different levels of IgG and IgA antibody production.^68^ Interestingly, antigen presentation is also more critical in dictating the cellular uptake of nanoparticle vaccines than the presence of targeting ligands, such as mannose.^69^

Significant efforts have also been dedicated to the targeting of nanoparticle vaccines to the lymphoid organs. Transport to the lymph nodes is essential for antigen presentation leading to T‐cell activation, which leads to cytotoxic T‐cell responses and B‐cell activation that stimulates the production of high affinity antibodies.^70^ Nanoparticle size has been identified as a critical parameter that influences targeting to the lymph node. Evaluation of 20, 45, and 100 nm PPS nanoparticles administered intradermally demonstrated an inverse correlation between lymph node retention and nanoparticle size. Moreover, a targeting ligand was not necessary for significant accumulation of the 20‐nm nanoparticles to the lymph nodes.^71^ The same preference for smaller sized nanoparticles was observed after intravascular administration—30‐nm polymeric micelles were found to extravasate and accumulate at metastatic lymph nodes more than 70‐nm micelles of similar chemical composition.^72^

As an alternative strategy, nanoparticles can also be preloaded into dendritic cells, which can home to the lymph nodes after injection. In this situation, the optimal nanoparticle size maximizes dendritic cell uptake without inducing toxicity. Delivery of immune adjuvants (e.g., CpG) on the nanoparticles can enhance antigen copresentation and upregulate costimulatory molecule expression at the lymph nodes.^73^ To further enhance lymph node homing, nanoparticles can be coated with lipid membranes with incorporated ganglioside GM3. This facilitates interaction with Siglec1 on myeloid dendritic cells and macrophages, which is responsible for B‐cell, CD8+ T‐cell, and iNKT priming and activation.^74^ To evaluate the localization and biodistribution of nanoparticle vaccines, evaluation of trafficking to the lymph nodes can be conducted in vivo by magnetic resonance imaging (MRI) or single‐photon emission computed tomography (SPECT) with iron oxide or radioisotope labeling, respectively.^75^

#### 3.2.2 Enhancement of the antitumor immune response

**Figure 5:**
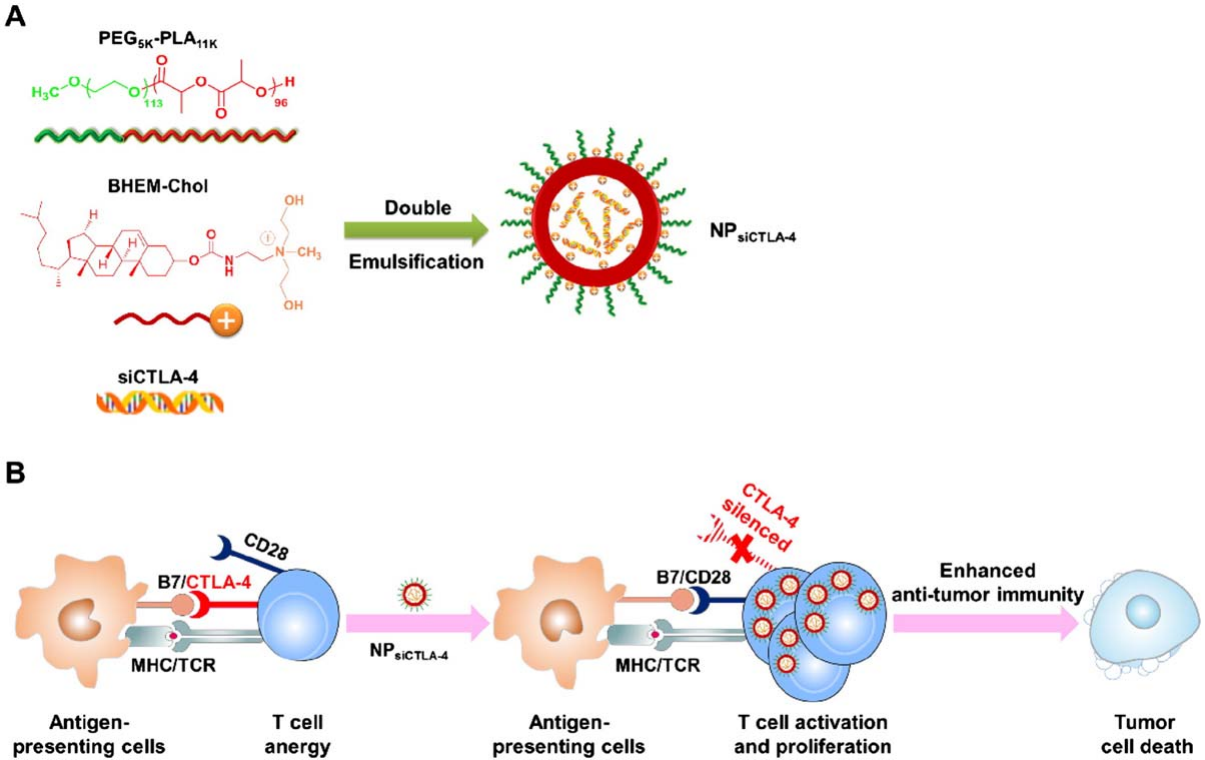
Nanoparticles to treat immune suppression. (A) Preparation of siCTLA‐4‐encapsulated nanoparticles (NP_siCTLA‐4_) with poly(ethylene glycol)‐*block*‐poly(d,l‐lactide) and a cationic lipid BHEM‐Chol by double emulsification. (B) Enhancing T‐cell‐mediated immune responses by blocking CTLA‐4 using NP_siCTLA‐4_. CTLA‐4 plays a strong inhibitory role in T‐cell activation and proliferation, which significantly curbs T‐cell‐mediated tumor rejection. NP_siCTLA‐4_‐mediated CTLA‐4 knockdown enhanced the activation and proliferation of T‐cells, which inhibited the overall growth of tumors. Reproduced with permission from ref. 76

In addition to serving as tumor vaccines, nanoparticles favorably modulate the immune response through multiple means. One challenge with cancer immunotherapy is the presence of immune suppression, which mutes the immune response in the tumor microenvironment. An example of an immune suppression mechanism is checkpoint blockade, in which tumor cells upregulate ligands that bind to T‐cell receptors (e.g., PD‐1) that downregulate cytolytic activity. Nanoparticle immunotherapies have been designed to interfere with this immune suppression mechanism. In a melanoma model, polymeric nanoparticles loaded with cytotoxic T lymphocyte‐associated molecule 4 (CTLA‐4) siRNA have been able to increase the number of antitumor CD8+ T‐cells while simultaneously decrease the number of regulatory T‐cells (T‐regs) (Figure 5).^76^ The delivery of immune adjuvants by PPS nanoparticles to dendritic cells in the draining lymph node also increases the CD8 to CD4 T‐cell ratio, which also leads to slowed tumor growth in a melanoma model.^77^ PPS nanoparticles functionalized with exposed hydroxyl groups also can engage the complement system, which is indicated by high C3a release from the antibody‐antigen complex.^78^ Hyaluronan nanoparticles have the unique property of initiating an innate immune response upon interaction with CD44, which is a tumor‐specific marker in some forms of leukemia.^79^

**Figure 6:**
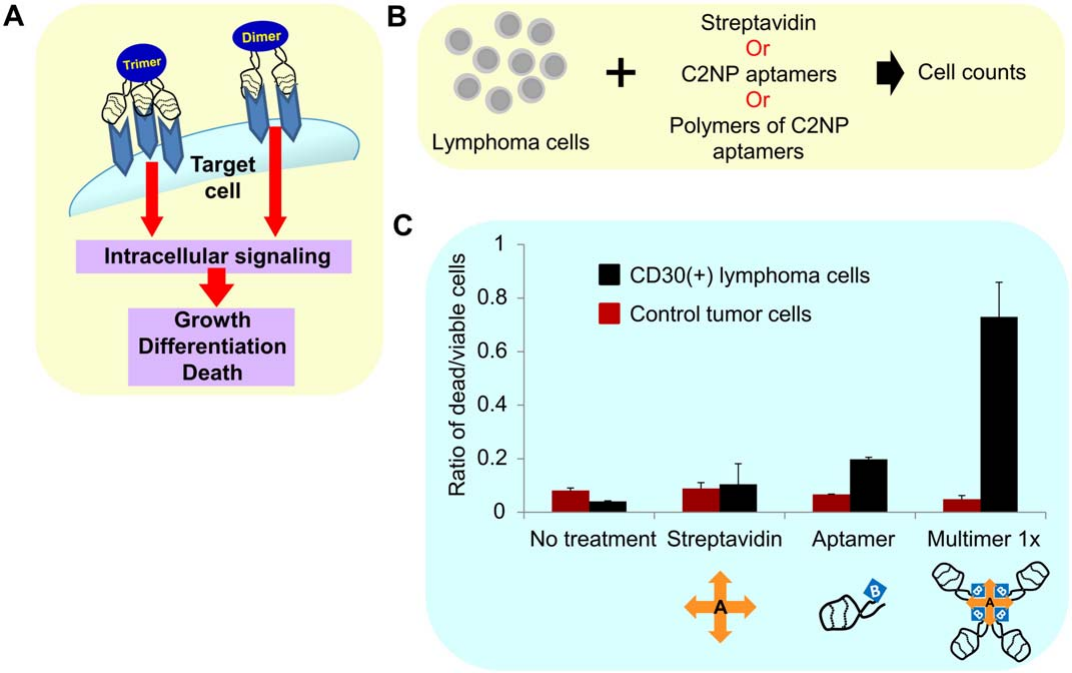
Targeted multivalent aptamers deliver biotherapy to anaplastic large cell lymphoma. (A) Schema showing receptor oligomerization inducing downstream signaling. CD30‐associated signaling is activated by its ligand through trimerization of the receptor, leading to varied outcomes that range from apoptosis to proliferation. (B) CD30‐positive and ‐negative cells were incubated without any treatment; in presence of control streptavidin, monomeric aptamer C2NP, and multimeric aptamer C2NP, for 72 hr to detect aptamer‐mediated CD30 signal transduction. A multivalent CD30 aptamer was made using biotinylated C2NP (3×) with streptavidin (1×). (C) The multivalent CD30 aptamer‐induced signaling, resulting in a higher percentage of dead cells in CD30‐positive ALCL (K299 cells), and had no effect on cell death in CD30‐negative (HL60) cells. Reproduced with permission from ref. 81

To further boost therapeutic efficacy, it is advantageous to develop means to target tumor environments with higher specificity. A powerful “next generation” targeted nanoparticle for immunotherapy applications is the aptamer, which consists of single‐stranded RNA or DNA oligonucleotides that can form structures which have high affinity to their targets. An aptamer acting as an agonist to CD40, which enhances the immune response by promoting B‐cell clonal expansion and germinal center formation, increased the median survival of mice with A20 B‐cell lymphoma by approximately 10 days.^80^ Another ssDNA aptamer has been designed to target the CD30 receptor, which trimerizes to activate cellular signaling that triggers cell apoptosis in anaplastic large cell lymphoma (Figure 6).^81^ Aptamers have also been used against targets in the tumor stroma. The versatility of aptamer design can facilitate the pursuit of multiple targets; in a breast cancer study, it was shown that the targeting of both the VEGF receptor and the 4‐1BB receptor, a costimulatory receptor that promotes the survival and activation of activated CD8+ cells, provided survival benefit over treatment against each target individually.^82^ Another innovative strategy exploits how the natural biodistribution of healthy lymphocytes mirrors the biodistribution of hard‐to‐reach tumors. By loading nanoparticles onto T‐cells, chemotherapies with poor pharmacokinetics have successfully been delivered to disperse lymphomas with significantly higher therapeutic effect than soluble drug or free nanoparticles.^83^

## 4 Conclusions

We return to the question posed at the beginning of the article, how do we deliver the optimal amount of immunotherapy to a specific site, with appropriate kinetics and dosing schedule, without inducing deleterious side effects that outweigh the benefits of the therapy? Nanoparticles are an appealing solution to this complicated drug delivery problem. They are versatile, engineered platforms that can be tailored to home to specific targets. By fine‐tuning nanoparticle size, shape, and elasticity, nanoparticles can be guided to travel to a specific site. To decrease accumulation in off‐target sites and minimize toxicity that is a result of nonspecific localization, surface modifications can be implemented to target nanoparticles to specific cells and prevent uptake by other cells. The formulation of nanoparticles with stimuli‐sensitive materials (e.g., pH, enzymatic) or inside hydrogels and matrices enables immunotherapeutic release only when the particles have reached their target microenvironment. It is important to realize that a single design modification will likely not maximize site‐specific delivery, minimize off‐target accumulation, and optimize immunotherapy delivery timing, tissue penetration, and intracellular delivery. An ideal design will achieve a set of these goals and will be most effective when tailored specifically to its desired therapeutic use.

In the clinic, nanoparticles have been approved for the delivery of chemotherapy to cancer patients. Liposomal formulations loaded with chemotherapeutics such as doxorubicin, paclitaxel, and daunorubicin are recommended as neo‐adjuvant therapies in ovarian cancer, Kaposi's sarcoma, and multiple myeloma. At the same time, nanoparticles are under clinical evaluation for the delivery of therapeutic siRNA, the detection of angiogenesis and micrometastatic lesions, and for the measurement of cancer biomarkers in the blood. Nanoparticle immunotherapies, however, have not traveled as far along the research and development pipeline. For clinical adoption to become a reality, it is essential to improve the design of nanoparticle immunotherapies so their targeting specificity is enhanced. In addition, it is important to continue boosting the efficacy of nanoparticle immunotherapies under development to justify their use over existing therapeutic regimens. One avenue of research will be to continue the evaluation of new combinations of immunotherapies. When developing and testing combinatorial immunotherapies, it is important to realize that a set of nanoparticles of different designs, not a single particle, may be necessary to maximize therapeutic effect. Mechanistic studies should be combined with in silico studies to understand how the immune response evolves over time with the treatment and the disease. At the same time, it is important to consider the immunogenicity of the nanoparticle delivery vehicle in combination with the immunogenicity of the therapeutics they carry.

It is also essential to grasp how a nanoparticle immunotherapy affects immune homeostasis. While design modifications to a nanoparticle may enable site‐specific targeting and targeted delivery to specific cell compartments, these same modifications may disrupt cellular membranes, induce apoptosis, or interact with pattern recognition receptors and produce proinflammatory cytokines. The common approach to enhance cancer immunotherapy efficacy by reversing immune suppression could lead to increased T‐cell activation or proinflammatory cytokine generation in healthy tissues. In addition, therapeutic delivery above the optimal dose could potentially activate immune suppression mechanisms that were previously dormant. Immunotherapies for autoimmune diseases face the opposite challenge. While immune suppression could favorably reduce inflammation in host tissues, appropriate dosing must be used to prevent increased susceptibility to infectious diseases. For these endeavors to be successful, it is necessary to understand how nanoparticle immunotherapies perturb the complex network of signaling pathways involved in immunity. The ability to intelligently engineer nanoparticle immunotherapies will allow us to effectively attack new targets as they are discovered. Using nanoparticles, long‐term, synergistic, reactive immunotherapy regimens can be developed to keep diseases of immune dysregulation in check.
